# Doctors’ Conflict-of-Interest; What it Looks Like and What to do About it

**DOI:** 10.1177/27551938261431289

**Published:** 2026-03-19

**Authors:** Joel Lexchin

**Affiliations:** 1 School of Health Policy and Management, 7991 York University, Toronto, Canada; 2 Faculty of Medicine, University of Toronto, Toronto, Canada

**Keywords:** conflict-of-interest, disclosure, doctors, gifts, pharmaceutical companies, policies, restrictions

## Abstract

Conflict-of-interest (COI) between doctors and the pharmaceutical industry is widespread and is present in the majority of practitioners. The effects of COI on how doctors prescribe and hence on the health of their patients is negative. This article first summarizes the widespread nature of COI and then examines what forms it takes and how doctors view COI. It next looks at the two ways that have been used to try and limit its influence – managing COI and avoiding COI – and shows that managing COI by limiting gifts to small amounts and relying on declaring COI are based on false assumptions and have little chance of producing any positive results. It then goes on to review the measures that have been shown to be beneficial, particularly policies and legislation restricting or prohibiting certain types of COI among medical students, residents and practicing doctors. Finally, this article looks at how to build upon the successes that have occurred and what more could be done.

## Introduction

Financial conflicts-of-interest (COI) between individual doctors and drug and device makers (hereafter drug companies) are widespread. The best data come from the United States (US) where over 57% of doctors received payments from drug companies from 2013 to 2022.^1^ Their COI is often hidden as can be seen by looking at the relationship between principal investigators and randomized controlled trial (RCT) outcomes, who writes articles promoting the off-label use of medicines, COI among guideline writers, industry payments to oncologists and who advocates for the use of controversial medicines.

The widespread nature of COI between individual doctors and companies has prompted multiple different approaches to try and ensure that the consequences of interactions do not adversely affect the health of patients and, secondarily, do not lead to unnecessary increases in the cost of drug therapy. On a simplistic level, these strategies can be divided into those that allow COI to continue to exist and then attempt to manage it versus those whose aim is to restrict COI in the first place. This article will first use illustrative examples from the international literature to describe the level of COI that exists between doctors and industry and then will deal with the success of managing and restricting COI in mitigating its consequences. It then discusses the importance of strong leadership in the medical profession in minimizing the level of COI. Finally, I will offer recommendations about how to move forward with controlling and minimizing COI.

This article is not a systematic review that gathered and summarized the large volume of literature on the topic of doctors’ COI and how to minimize the effect of that COI. Rather, it is a narrative review that draws on my 45-year background of researching and writing about this topic, my familiarity with the literature as well as a close examination of two recent key scoping reviews; one identified all known ties between the medical product industry and the healthcare ecosystem,^2^ the second gathered literature about interventions for managing physician-pharmaceutical industry interactions.^3^

## COI is Everywhere

Ahn and colleagues examined financial ties between principal investigators and pharmaceutical companies in 132 studies.^4^ Two-hundred and thirty-one of 397 principal investigators had financial ties with those ties being present among 103 investigators where the study was positive and just 29 where the study was negative. A financial tie was significantly associated with a positive randomized controlled trial outcome with an odds ratio of 3.57.

When it comes to off-label marketing activities, only one in seven authors identified in whistleblower complaints adequately disclosed their conflict of interest in subsequent journal publications.^5^

Among 37 studies including 14 764 total guideline authors, 45% had at least one COI, with the prevalence ranging from 6% to 100%.^6^ Eight studies reported the average payments per author and they ranged from $578 to $242 300. (All dollar amounts are in US dollars.) Among the 10 studies that included data on undisclosed COI, 32% of authors had undisclosed industry payments. COI among authors of editions of the Diagnostic and Statistical Manual of Mental Disorders has been extensively studied. For the fifth edition, published in 2022, there were 92 panel or task force members who were physicians based in the United States (US) and who therefore had entries in the Open Payments database.^7^ Fifty-five (60%) of these 92 panelists received payments from industry.

During 2016–2018, industry made general payments to 52 441 US physicians for 137 unique cancer drugs.^8^ The annual number of payments and the total value increased over the study period by 20% and 31%, respectively. In 2018, 5% of physicians (n = 1660) received *>*$10 000 in annual payments and 0.6% (n = 209) received *>*$100 000.

Clinicians treating cancer made 261 submissions to the pan-Canadian Oncology Drug Review about whether new oncology drugs should be publicly funded. Financial conflicts with drug companies were declared in 176 (66.3%) of all submissions.^9^ In 21 (45.7%) of submissions for 46 drug indications, 50% or more of the clinicians had a conflict with the company making the drug.

Controversial drugs that generate large amounts of revenue for the companies selling them are often defended by physicians with COI. Authors who supported the use of calcium-channel antagonists in the debate over their safety in the treatment of cardiovascular disorders were significantly more likely than neutral or critical authors to have financial relationships with manufacturers of these drugs – 96%, versus 60% and 37%, respectively.^10^ Whether there are harmful effects of varenicline, a treatment for smoking cessation, has been highly contested. Authors of opinion pieces who minimized the cardiovascular and psychiatric risks were more likely to report financial ties to the pharmaceutical industry compared to those without COI with an odds ratio of 4.00 for cardiovascular risk and an odds ratio of 3.79 for psychiatric risk.^11^

In the wake of the Women's Health Initiative (WHI) study that found that the risks of menopausal hormone therapy (MHT) outweighed the benefit for asymptomatic women, there was a vigorous defense of MHT mounted and considerable criticism of the study. One analysis looked at 114 articles (58 editorials, 16 guidelines, 37 reviews, 3 letters), of which 110 (96%) had a partisan stance favoring MRT. “All five prolific authors disclosed in at least one of their articles that they had been funded by industry or that they had been related to some potential conflict of interest. However, these relationships with hormone manufacturers were revealed in only 6 of their 110 partisan articles; in the other 104 articles, no such disclosure was stated”^12^ (page 917). A second article analyzed the tone in opinion pieces on hormone therapy published in the first four years after the estrogen-progestin arm of the WHI was stopped. Of the ten authors studied, eight had declared payment for speaking or consulting on behalf of menopausal hormone manufacturers or for research support from them.^13^ Thirty of 32 articles (90%) evaluated as promoting MHT were authored by those with potential financial conflicts of interest, compared to 11 of 18 articles (61%) by those without such conflicts.

Further evidence of the widespread nature of COI comes from looking at payments received by doctors in multiple countries. From 2013 to 2022, 826 313 (57.1%) of 1,445,944 eligible physicians in the US received one or more payments from drug companies.^1^ Payments (cash and other transfers of value, ie, non-cash) were for a wide range of reasons including consulting services, non-consulting services (such as fees for serving as a speaker or faculty at a venue), food and beverages, travels and lodging, entertainment, education, gifts, grants, charitable contributions and honoraria. The percent of doctors who had interactions with companies does not include those who accepted drug samples from companies. According to a 2011 survey 77% of US doctors used samples.^14^ A more recent 2017/2018 survey found that just under 60% of practices delivering primary care had a “free sample closet”.^15^ There is undoubtably an overlap between doctors accepting payments and free samples, but the percent of US physicians with a COI at any one time is likely to be greater 60%.

A national survey of 3167 US physicians in six specialties (anesthesiology, cardiology, family practice, general surgery, internal medicine, and pediatrics) in late 2003 and early 2004 found that 94% reported some type of relationship with the pharmaceutical industry. Most of these relationships involved receiving food in the workplace (83%) or drug samples (78%). More than one third of the respondents (35%) received reimbursement for costs associated with professional meetings or continuing medical education, and more than one quarter (28%) received payments for consulting, giving lectures, or enrolling patients in trials.^16^

In Canada, in a 2015 survey of just over 1000 doctors, 46% said that they had been retained by a drug company at some point in their career.^17^ The figure of 4.9% of Australian doctors who received a transfer of value from a drug company during November 2019 to October 2022 may seem low but it does not include payments from companies that are not members of Medicines Australia, the main industry lobby group, and it excludes physicians who received meals and beverages that were offered at over 90% of company-sponsored events.^18^

## Approaches to Dealing with COI

### Managing COI

#### Limiting Transfers of Value to Small Amounts

The belief that only large payments will affect the behaviour of doctors is common and has led to policies that allow them to accept smaller amounts. For example, the marketing codes of both PhRMA and Medicines Australia allow companies to offer meals to doctors provided that they are only of “modest” value.^19,20^

However, the idea that small payments do not have an effect is based on the false premise that doctors consciously make decisions to base their actions on their COI. The reality is that reciprocating for the receipt of a gift is largely an unconscious act. Robert Cialdini, the Regents’ Professor Emeritus of Psychology and Marketing at Arizona State University, looked at the behaviour of passengers waiting in an airport when they were approached by a robed member of the Hare Krishna Society offering them some item of small value such as a flower. “Often, people attempted to return the gift, but the Krishnas refused to take them back, requesting a donation instead. People who didn’t want the flowers often gave money anyway”^21, p. 20)^.

Residents who were initially reluctant to accept gifts from companies (only 21.7% found them appropriate) were then provided with reminders of the sacrifices that they had made to date in their careers, including the hours that they had worked, the number of hours they were able to sleep, their salary, and their education-related debt. Next, they were provided with implicit rationalizations for ethically questionable behaviour. At that point, their willingness to receive gifts increased to over 60%, despite the fact that the majority disagreed with the suggested rationalization.^22^

An experimen­tal study conducted by Ulrike Malmendier and Klaus Schmidt showed that small gifts strongly affected recipients’ decisions in favour of the gift-giver even though the recipients were aware that the gift was given with the inten­tion of influencing their behaviour.^23^

The association between small gifts and attitudes of recipients about both drugs and prescribing decisions has been demonstrated in a couple of studies. In one randomized trial, third- and fourth-year medical students at two US medical schools were were exposed to small branded promotional items for Lipitor (atorvastatin) and then their attitudes toward Liptor were compared to their attitudes about a competing cholesterol lowering agent, Zocor (simvastatin). Students at one medical school without restrictive policies limiting pharmaceutical marketing had more favourable implicit attitudes about Lipitor compared to Zocor. At the second medical school that had such policies the effect was reversed.^24^

In the second study, prescribing behaviour for four classes of medications – statins, cardioselective β-blockers, angiotensin-converting enzyme inhibitors and angiotensin-receptor blockers and selective serotonin and serotonin-norepinephrine reuptake inhibitors – was examined in US physicians who received meals worth $20 or less from companies promoting the most-prescribed brand-name drug in each class. In all four cases, there was an increased rate of prescribing the promoted medication compared with alternatives in the same class.^25^

#### Declaring a COI

Declaring a COI in journal articles and talks has become a de facto standard in an effort to increase transparency about individual COI on the supposed grounds that when people read an article or hear a talk, they can take the COI into account when evaluating what the writer or speaker are saying. This supposition about the value of transparency rests on shaky grounds. As Jerome Kassirer, a former editor-in-chief of the New England Journal of Medicine, notes “it is difficult for anyone who is not a highly specialized domain expert to identify a biased opinion in spoken or written material…Disclosure requires people to assess a physician's motives and guess whether his or her actions may have been affected by any conflicts”^26, p. 86)^.

Furthermore, there are two major psychological mechanisms that mean that disclosure can have the unintended consequence of producing a greater degree of bias. Strategic exaggeration is the tendency of advisors to inflate the bias in their speeches or writing to counteract any discounting that might occur because of their disclosure. For example, a doctor giving a talk could compensate for anticipated discounting by “playing up” the benefits of a new drug. The second mechanism is moral licensing which refers to the undermining of professionalism that can occur as a result of disclosure. Experimental research suggests that after engaging in moral behaviour people feel “licensed” to act immorally in subsequent interactions.^27^ In addition, having heard a disclosure of COI, the audience may be inclined to view the speaker or writer as someone who is “honest” and therefore trustworthy and discount any potential biased information from that person.^26^

There is also considerable evidence that the authors of journal articles and clinical practice guidelines only provide partial disclosures or fail to disclose at all, as has already been briefly mentioned. Among 270 US physician authors of 20 clinical practice guidelines published in 2020 72 (26.7%) of the authors accurately disclosed their financial COIs, including 68 (25.2%) accurately disclosing no financial COIs and 4 (1.5%) accurately disclosing a financial COI. In contrast, 101 (37.4%) disclosed no financial COIs and were found to have received payments from industry.^28^

COI was examined in 100 trials published from February 2011 to May 2013 that included 318 Danish non-industry employed authors. There were undisclosed COIs for 40 of 318 authors related to the trial sponsor or manufacturer of trial drugs. Twenty-five percent of the authors had undisclosed COIs related to competing companies manufacturing drugs for the same indication and 43% had undisclosed COIs with any drug manufacturer.^29^

A systematic review of studies that compared individual reporting of COI with objective data sources reporting payments from industry found that the pooled proportion of COI discrepancies at the article level was 81%, 79% at the payment level and 66% at the author level.^30^

Finally, slides with oral presentations of speakers’ COI are displayed at conferences for a median of just 2 s and less than a third of presenters discussed their conflict after projecting their slides.^31^

#### Payment Databases

Declarations of COI at an individual level have been complemented by databases that record payments from drug companies on a systematic basis. The majority of databases are maintained by national pharmaceutical associations^32^ and many allow for physicians to opt out from disclosing payments to them.^33^ For example, in the United Kingdom (UK) the estimate is that only 55% of transfers of value are reported at an individual level.^34^ The major exceptions to this pattern are the government legislated company disclosure policies in France, Portugal and the US.^35^ In the US, the Open Payments database maintained by the Centers for Medicare and Medicaid Services records all transfers of value over $10 to US doctors. The database started reporting in August 2013 and to date (June 2025) has published over 15 million records of transfers of value.^36^

An analysis of the effect of earlier US state level databases found that, contrary to expectations that disclosure would change practice, there was very little switching from brand-name to generic therapies and what switching that did occur was not statistically significant.^37^ Although the advent of the Open Payments database has been a boon to people examining the effects of COI on prescribing behaviour, the evidence about whether the transparency that it promotes has affected payments is mixed and the evidence that it has affected how doctors prescribe is absent. In the first 5 years after Open Payments came into effect, contributions to doctors from 20 top-spending medical technology companies collectively more than tripled.^38^ More generally, between 2014–2018 the proportion of physicians receiving industry payments decreased across all specialties, while the total value of payments remained stable except for a decrease in primary care physicians. Annual payment values decreased for physicians receiving less than $50 000 whereas physicians receiving more than that amount continued to receive similar or greater amounts.^39^ In the ophthalmology community, increasing requirements for disclosure of financial relationships by participants at the annual meeting of the American Academy of Ophthalmology between 2008 and 2015 was not associated with a decrease in financial disclosures associated with potentially beneficial physician-industry ties, although the number of ethically questionable disclosures may have decreased. In addition, the percentage of participants with at least one financial disclosure and the mean number of disclosures per participant increased over the time period, indicating that disclosure may have had a permissive effect, i.e., physicians felt more inclined to have industry relationships.^40^

The most recent analysis of the effect of the Open Payments database shows that payments for meals has decreased across a 10 state region in the US whereas the picture is more nuanced for travel related payments.^41^ The significance of the changes in payments is unclear.

### Avoiding COI

#### Local and Regional Initiatives

There have been multiple local efforts to try and restrict interactions between practicing doctors and drug companies. What follows is just a sample of the initiatives that have been taken. Doctors and their staff at the Kelsey-Seybold clinics in the Houston area stopped receiving free breakfasts and lunches after the clinics barred pharmaceutical representatives from visiting doctors unless a doctor specifically requested an appointment.^42^ The Cleveland Clinic publicly reports the business relationships that any of its 1800 staff doctors and scientists have with drug and device makers.^43^ ProHealth which has 90 practice locations in Connecticut and cares for more than 10% of the state's population does not allow visits by sales representatives and the Wheeler Clinic, which provides medical care to residents in over 15 communities in Connecticut does not allow physicians to accept samples or interact with sales representatives.^44^ The 100-doctor group at Providence Medical Group in Portland Oregon has banned free lunches and all visits from sales representatives.^45^ The 5-doctor Madras Medical Group in rural Oregon, which employs 18 support staff, stopped accepting and distributing drug samples and discontinued seeing sales representatives. “Provider champions” at the clinic educated themselves and other clinicians on the literature related to the ethics of physician-industry relationships and their effect on practice. In order to replace the meals, gifts and information that the representatives provided, the practice implemented a monthly all-clinic lunch with protected time for staff, it disposed of all drug-branded office supplies and non-industry information sources were identified.^46^ The Kaiser Permanente Medical Group strongly discourages its 7000 doctors from accepting free drug samples from sales representatives.^47^

Outside the US, the 12 doctors at a family medicine teaching unit associated with St. Michael's Hospital in Toronto, unanimously agreed to stop accepting drug samples from companies.^48^ The Inala Health Centre General Practice situated in a socially disadvantaged part of Brisbane, Australia is an academic general practice with seven part-time general practitioners, three practice nurses, three regular reception staff and a practice manager. The staff there adopted a policy of reduced access to sales representatives including reception staff not making appointments for doctors to see them and not accepting promotional material; sales representatives cannot access sample cupboards and any doctor wanting to see a sales representative has to do so outside clinic hours. Before the policy was adopted, there were 239 items of promotional material in the practice and 4660 tablets in the sample cupboard. These were reduced by 32% and 59%, respectively, at 3 months after the policy was adopted and the reduction was sustained at 9 months. Generic prescribing increased significantly at 3 months and 9 months.^49^

Aside from the limited evaluation in the Australian clinic there does not appear to have been any other formal evaluation of the effects on prescribing behaviour of the adoption of the various local policies.

### Education and Exposure to Restrictive Institutional Policies

Healthcare and educational institutions have adopted policies about COI for faculty, initiated programs to teach medical students and residents about COI and used policies to restrict interactions between medical students and residents and pharmaceutical companies, all in an attempt to help learners and clinicians to avoid COI. Many major medical centres including Yale, Stanford, Penn, and the medical campuses of University of California at Los Angeles and Davis have outlawed small gifts.^26^ The University of California, Davis forbids its medical staff from accepting any gifts from drug salesmen, including drug samples, pens, mugs, and meals.^50^ The Penn Health System has stopped drug companies from giving out meals and company merchandise to doctors^51^ and Stanford University Medical Center prohibits its physicians from accepting even small gifts like pens and mugs.^52^

However, policies are only effective if faculty are aware of them and the policies are enforced. When clinical researchers at Stanford and University of California, San Francisco were interviewed about their institutions’ policies, fewer than half could describe them.^53^ Moreover, actual practices often differed from what is outlined in policies. Many institutions used processes for reporting and managing conflicts of interest that were different than the processes described in their policies.^54^ There does not appear to be any literature about whether policies are systematically enforced.

There have been two systematic reviews that analyzed curricula for medical students and residents regarding COI with drug companies. One, done in 2008, examined 9 curricula and found modest improvements in resident confidence, knowledge of guidelines, belief in the potential influence of marketing on behaviour, and self-reported acceptance of gifts. However, no studies included long-term follow-up to determine if the changes persisted.^55^ The second review, nine years later, found 20 publications. Some publications described a change of attitudes toward a stronger skepticism regarding interactions with pharmaceutical companies. Four publications described improved knowledge, one publication described a change in behaviour toward a reduction of the acceptance of gifts. Once again there were no data on the sustainability of the courses’ effects on participants’ behaviour.^56^

The degree of interaction with drug companies was compared among three groups of internal medicine residents in Canada after they were out in private practice. The first group were those who went through a residency program after the program instituted a policy restricting company access to them, the second were those who went through the residency program before the policy was started and the third was a group who went through a residency program at another university without a comparable program. Compared to the latter two groups, those who went through the residency program after the restrictive policy was instituted were less likely to find information from company representatives beneficial in guiding their practice. However, all three groups were equally likely to report that they met with sales representatives in their office in the past year. Whether those affected by the policy had different prescribing practices was not measured.^57^

Two other studies that compared medical students and residents exposed to policies restricting interactions or gifts with those not exposed to those policies did evaluate prescribing behaviour once the doctors were out in private practice. One study looked at prescribing of newly marketed products in three psychotropic classes of drugs, none of which represented radical breakthroughs in therapy. For two of the three medications, attending a medical school with an active gift restriction policy was associated with reduced prescribing of the newly marketed drug. Among cohorts of students who had a longer exposure to the policy or were exposed to more stringent policies, prescribing rates were further reduced.^58^ The second study analyzed whether exposure to COI policies of varying degrees of restrictiveness during psychiatric residency training affected antidepressant prescribing after graduation. Rates of prescribing of heavily promoted drugs were lower among post-COI graduates than pre-COI graduates at all levels of COI restrictiveness, with differences being larger for those exposed to more restrictive policies.^59^

### Legislated Policies in US States

Several states in the US have adopted policies to try and deal with COI. King and Bearman described the legislative attempts to control COI as of 2017 and what follows comes from their analysis.^60^ Vermont, Massachusetts and Minnesota banned most gifts to physicians and had the most comprehensive disclosure requirements for non-prohibited payments. Maine, West Virginia, and Washington D.C. required companies to report aggregated marketing expenditures to the state, but the disclosure data from these three jurisdictions were not readily available via public websites. California and Nevada mandated that pharmaceutical companies adopt and comply with the guidelines developed by the Pharmaceutical Research and Manufactures of America (PhRMA). PhRMA's Code prohibits entertainment and recreational items, as well as gifts not related to patient care or education. The guidelines do allow for meals accompanied by educational presentations and discussions, as well as educational gifts of $100 or less per item. Payments or gifts that fall outside of the guidelines do not have to be disclosed.

Using a dataset that captured 189 million psychotropic prescriptions written between 2005 and 2009 for Vyvanse™ (a stimulant), Invega™ (an antipsychotic), Pristiq™ (an antidepressant) and Cymbalta™ (an antidepressant), the authors found that uptake of new costly medications was significantly lower in states with marketing regulations than in areas that allowed unrestricted pharmaceutical marketing. In states with gift bans, they observed reductions in market shares ranging from 39% to 83%. Policies banning or restricting gifts were associated with the largest reductions in uptake. Disclosure policies were associated with a significantly smaller reduction in prescribing than gift bans and gift restrictions.^60^

### Policies from Organizations That Set or Influence Professional Standards

Between April 2021 and November 2021, Brown and colleagues^61^ studied the COI policies of the five largest UK Royal Medical Colleges, the equivalent Medical Colleges in Australia, New Zealand and Canada, the medical regulators in these countries, the American Academy of Family Physicians, the British Medical Association, eight NHS Trusts and seven Clinical Commissioning Groups (CCGs). (CCGs, until they were abolished in July 2022, were UK clinically-lead statutory bodies that were responsible for assessing local needs, deciding priorities and strategies, and then buying services on behalf of the population from providers such as hospitals, clinics, community health bodies.) Two of the 34 organizations had no policy on the management of COI and one did not respond to two invitations to take part in the study. Twenty-nine of the 31 policies that were evaluated “referred to the need for individuals to apply judgement when deciding whether an interest is a conflict, with just over half (18/31) advocating a low threshold. Policies differed on the perception of frequency of COI, the timings of declarations, the type of interests that needed to be declared, and how COI and policy breaches should be managed. Just 14/31 policies stated a duty to report concerns in relation to COI”^61, p. 295)^. Overall, the authors concluded that there was a wide variation in what interests should be declared, when and how. The authors did not comment on whether the policies were being enforced or the effectiveness of any of the policies that had been evaluated.

The situation with the College of Physicians and Surgeons of Ontario (CPSO), the licensing and disciplinary body for doctors in the Canadian province of Ontario which initially adopted a policy in 1992 and subsequently updated it several times, most recently in March 2024, is similar.^62^ Although the policy has multiple statements about what physicians must do and must not do, there is no statement about how the CPSO will enforce its policy, nor is there any record since it adopted its first policy that the policy has ever been enforced.

Other types of organizations that were not included in the Brown study such as medical societies and associations also have adopted policies. For example, the Wisconsin Medical Society has adopted a policy that advocates that physicians should not accept any gifts “from any provider of products that they prescribe to their patients such as personal items, office supplies, food, travel and time costs, or payment for participation in online CME”^63, p. 96)^. There was no explicit rationale mentioned for this recommendation. The Canadian Medical Association updated its guidelines on interactions with industry in 2021.^64^ The guidelines are explicit that physicians “may not always be aware of, or able to accurately self-assess, how their industry affiliations can subconsciously influence their judgment, their assessment or presentation of medical evidence, their clinical decisions and their prescribing”^64, p. 1)^. However, medical associations and societies are voluntary membership organizations which means that regardless of how strong (or weak) their policies may be, they have no means of enforcing them beyond moral suasion.

### Deep Professionalism

Medical students, residents and practicing doctors exhibit cognitive dissonance when it comes to their vulnerability to interactions with industry. They are willing to admit that their colleagues may be influenced but think that they themselves are largely invulnerable even as they also acknowledge that messaging from industry is likely to be biased. This self-deception has been shown in multiple studies in different countries.^65–68^ In what is probably the most widely cited study of this phenomenon, Steinman and colleagues surveyed first- and second-year US internal medicine residents about their attitudes and behaviours towards industry gifts.^69^ Sixty-two (61%) of the residents felt that their own behaviour was immune from influence but only 15 (16%) believed the same to be true of their fellow residents. Despite, these beliefs, 80 (82%) considered interactions with pharmaceutical representatives appropriate for residents.

This deep-seated self-denial is one the factors behind Sah's efforts to promote what she refers to as “deep professionalism”.^70^ Sah starts from the point of view that people with a high sense of professionalism may be more vulnerable to COI as a view of oneself of being invulnerable to bias can lead to less effort to scrutinize one's decisions or behaviours for potential bias. Sah calls for “professionalism to be redefined as a deeper concept that includes an understanding of the limitations of self-regulation and a set of observable repeated behavioral practices rather than a character trait”^70, p. 897)^. She contrasts this view of professionalism with the modern notion of the belief in the “ability to *self-regulate* and make *ethical, objective, impartial,* and *independent decisions* in the *interest of relevant others* [emphasis in original]”^70, p. 897)^. This view of professionalism arises from a feeling that succumbing to COI is a deliberate, conscious choice. As a result, physicians “often argue against external controls, such as rules and monitoring, on the grounds that their integrity and professionalism guarantee unbiased objective decisions even in the presence of such conflicts”^70, p. 903)^.

In contrast, Sah argues that deep professionalism in medicine requires mentors who act in an ethical manner combined with external rules that advocate for the avoidance of COI and a recognition of the limits of self-regulation, such that people understand that they may have a bias that they are unaware of and that they are vulnerable to that bias.

However, tempting as the idea of deep professionalism is, Sah only provides a high-level outline of how to achieve this objective and does not discuss the inevitable obstacles to achieving her remedies. She advocates for professional associations to “adopt programs such as the medical ethics morbidity and mortality rounds to devote more time to discussing cases with relevant ethical challenges and encourage professionals to articulate their doubts about managing their biases. Professional associations could also require continuing education to reinforce the concepts and maintain deep professionalism in organizations and society”^70, p. 910)^.

### Leadership

Leadership is a key element in the success of dealing with COI whatever the approach, but unfortunately it is often missing. Out of 161 US deans investigated in 2009, 9 were directors of 13 public companies, with 2 deans being directors of 2 companies and 1 dean being a director of 4 companies.^71^ In another study, 19 of 47 pharmaceutical companies had at least 1 board member who concurrently held a leadership position at an US academic medical center.^72^ Directors of 180 US healthcare companies were affiliated with 85 non-profit academic institutions, including 19 of the top 20 National Institute of Health funded medical schools. Included among the directors were eight medical school deans or presidents.^73^ Deans of Canadian medical schools are only a little better. Six out of 17 had COI that was often difficult to locate without considerable searching, but none of the deans held a leadership position (executive or board member) in a Canadian healthcare company.^74^

Forty-eight of 197 leaders of Australian diabetes or cardiovascular associations, and general associations serving doctors who manage these conditions received payments from companies, predominantly for speaker (51.4%) and advisory board (25.3%) engagements.^75^ Moynihan et al investigate the nature and extent of financial relationships between 328 leaders of US professional associations representing doctors working in the 10 costliest disease areas in the US and drug companies.^76^ His group looked at the proportion of leaders with financial ties to industry in the year of leadership, the four years before and the year after board membership, and the nature and extent of these financial relationships. Among 293 leaders who were medical doctors or doctors of osteopathy, 235 (80%) had ties. Total payments for 2017–19 leadership were almost $130 million with a median amount for each leader of $31 805.

### What are the Next Steps?

There are a number of points that need to be recognized when talking about solutions. First, as long as industry continues to be involved in the drug ecosystem, there will continue to be COI in medical practice. Second, while this article only deals with COI at an individual level, it is equally important to tackle the problem of institutional and medical society COI. Finally, no single reform measure is going to be successful in dealing with COI. It will take the coordination of multiple changes to have a substantial impact. What follows are some recommendations about how to move forward along with challenges to implementing them. Although these recommendations are a starting point more changes will be necessary.

The only measures that have been shown to reduce COI to date are legislated policies, specifically those policies that ban or restrict gifts to doctors, and policies that expose medical students and residents to restrictions about interactions with pharmaceutical companies. Measures should be taken to strengthen both by encouraging more jurisdictions and academic medical centres to enact strong policies. In the case of the US, to what degree medical schools are going to act on this measure is an open question since the American Medical Students Association stopped surveying and rating the COI policies of medical schools around 2017.^77^ In general, COI policies in medical schools and hospitals outside the US tend to be weaker than US ones, thus making it even more difficult to adopt the recommendations.^78^

The situation in France illustrates the difficulties that are likely to be encountered in getting national and subnational governments to adopt strong policies. The role of pharmaceutical sales representatives in France is regulated by the government through a Sales Visit Charter and a procedure certifying the conduct of sales representatives. The Sales Visit Charter is supposed to promote good practices such as the presentation of only approved product information including side effects and contraindications, but in reality key provisions within the Sales Visit Charter, such as mentioning adverse effects, precautions and contraindications are rarely respected.^79^ Sales representatives are not supposed to be allowed to provide content for continuing education courses. This function is supposed to be assumed by a national fund for continuing medical education that finances free courses for general practitioners and compensation for the loss of three days’ worth of medical income. The education is supposed to be given by impartial experts, but these experts can be opinion leaders with financial ties to the industry.

While transparency about payments on its own hasn’t been shown to affect COI and prescribing behaviour, it is still an essential element in combination with other measures in controlling COI. However, only a few European nations have strong public disclosure legislation about payments, with the rest relying on either industry self-regulation or a combination of industry and government.^32^ The province of Ontario's attempt to enact its version of Open Payments ground to a halt following the change in government in 2018.^80^ A federal body has to approve changes to the transparency code of Medicines Australia but the last time the code was revised, it was allowed to drop collecting data about payments for food and beverages.^81^ However, there are some small signs of possible change. One of the recommendations coming out of the inquiry in the UK into the 2020 independent review of medicines and medical devices safety was that “there should be mandatory reporting for pharmaceutical and medical device industries of payments made to teaching hospitals, research institutions, and individual clinicians.” Ninety percent of medical professional bodies surveyed by the BMJ agreed that there should be a mandatory and public register of doctors’ interests^82^ ^(page 1)^.

A Cochrane review on the effects of interactions between doctors and pharmaceutical companies on prescribing behavour that is just concluding, has identified studies with 93 results that deal with gifts and payments. The conclusion is that the overwhelming number of those results show that prescribing deteriorates on one or more of three measures – appropriateness, cost and quantity (Barbara Mintzes, personal communication, June 23, 2025). These findings make stronger control over promotion an urgent matter, but the current methods of dealing with promotion – industry self-regulation, quasi-independent third bodies or direct government regulation – all have serious deficiencies.^83,84^ What is needed is an independent body, established through legislation to give it the necessary authority to act decisively, with a membership composed of representatives of organizations that do not have COI, with the authority to levy meaningful sanctions and with stable, ongoing funding.^85^ The most meaningful sanctions, such as suspending all forms of promotion of a product for a period of time, could be used in cases where the promotion could reasonably be assume to have negatively affected patient health.

Finally, there is a need for strong leadership that will get behind these measures and keep pushing reform to the medical profession, to medical organizations and institutions, to the political leadership and to the general public. Ideally, leadership should come from the top down and from the bottom up. The positive reaction from the French National College of General Practice Teachers to the finding that gifts to general practitioners have a negative influence on prescribing shows that at least some medical organizations might be willing to take a leadership role in minimizing COI.^86^ Likewise, changes by groups of practicing doctors are an indication that local leadership may be developing.

## Conclusion

COI between doctors and companies affects the majority of doctors and degrades medical practice and the health of patients. Too many attempts to manage it are based on ideas that have no basis in fact and will do nothing to solve the problem. Measures such as legislation and medical school and residency policies that have been shown to have long-term effectiveness need to be adopted on a widespread scale and there should be intensive research into developing and evaluating other evidence-based ways of minimizing COI such as Sah's deep professionalism.

## Data Availability

All of the data used are available in the study.
